# Limewater

**Published:** 1896-04

**Authors:** 


### Limewater.

It is well known that limewater has a beneficial effect on the growth of children, and in countries where the drinking water is impregnated with salts of lime, the men are apt to be tall. An English medical authority states that for a perfect sanitary diet, alkaline water is needed for every person who eats heavily of meat, and this means nearly every one excepting the vegetarian.

